# Phenotyping of Obstructive Sleep Apnea Syndrome and Association with Cognitive Impairment, a Real-Life Study

**DOI:** 10.3390/biomedicines14061187

**Published:** 2026-05-24

**Authors:** Filippo Capilupi, Valentino Condoleo, Giandomenico Severini, Giuseppe Armentaro, Corrado Pelaia, Ilaria Gareri, Pasquale Loiacono, Maria Rosangela Scarcelli, Francesco Maruca, Alberto Panza, Marilisa Panza, Sofia Miceli, Raffaele Maio, Angela Sciacqua

**Affiliations:** 1 Department of Medical and Surgical Sciences, University Magna Græcia of Catanzaro, 88100 Catanzaro, Italy; 2 Geriatrics Division, “Renato Dulbecco” University Hospital of Catanzaro, 88100 Catanzaro, Italy; condoleovalentino@gmail.com (V.C.); raf_maio@yahoo.it (R.M.); 3 Department of Health Sciences, University Magna Græcia of Catanzaro, 88100 Catanzaro, Italy

**Keywords:** obstructive sleep apnea, phenotyping, cluster analysis, cognitive impairment, elderly, aging

## Abstract

**Introduction:** Obstructive sleep apnea (OSA) is highly prevalent, affecting up to 50% of individuals over 65 years. Elderly patients often present with atypical, fewer and less severe symptoms, suggesting age-specific phenotypes. However, comprehensive clinical phenotyping that incorporates cognitive outcomes remains limited. This study aimed to characterize OSA phenotypes through cluster analysis and evaluate their association with cognitive impairment, independently of age. **Materials and Methods:** Between 2020 and 2024, 409 adults with moderate-to-severe OSA were enrolled and stratified into three age groups (<65, 65–74, ≥75 years). All underwent home sleep apnea testing (HSAT), comprehensive symptom assessment, Epworth Sleepiness Scale (ESS), and Montreal Cognitive Assessment (MoCA, pathological ≤ 25 pts). Hierarchical cluster analysis (Ward’s method) used AHI, T90, BMI, and ESS. Logistic regression identified independent predictors of cognitive impairment. **Results:** Older groups showed lower BMI, higher comorbidity burden, fewer symptoms, and greater cognitive impairment prevalence (4.5% vs. 9.7% vs. 45.9%; *p* < 0.001), despite comparable polysomnographic severity across age groups. Cluster analysis identified three phenotypes: Cluster 1 (classical OSA: high AHI, BMI, T90, ESS); Cluster 2 (geriatric phenotype: low AHI, BMI, T90, ESS, highest cognitive impairment rate: 27.7%); Cluster 3 (hypersymptomatic: low AHI and T90, high sleepiness and asthenia, prevalent depression). On multivariate regression, age (OR 1.155; *p* < 0.001), male sex (OR 2.223; *p* = 0.034), and Cluster 2 (OR 3.131; *p* < 0.001) were independent predictors of cognitive impairment. **Conclusions:** Three clinically distinct OSA phenotypes were identified regardless of age and severity. The geriatric phenotype was associated with three-fold increased risk of cognitive impairment, supporting routine cognitive screening and age-adapted diagnostic strategies in elderly OSA patients.

## 1. Introduction

Obstructive sleep apnea (OSA) is a multifaceted disease characterized by recurrent episodes of partial or complete upper airway collapse, resulting in airflow cessation or reduction, chronic intermittent hypoxemia (CIH), increased production of reactive oxygen species (ROS), sympathetic hyperactivation, and sleep fragmentation. OSA, through these pathophysiological mechanisms, confers an augmented risk of cardiovascular, metabolic, neurological and oncological diseases [1]. It is the most common sleep-related breathing disorder affecting approximately 23.4% of women and 49.7% of men in a middle-aged population-based study [2]. Its prevalence further increases in the elderly population, affecting over 50% of individuals aged over 65 years [3]. The pathogenesis of OSA is multifactorial and results from the interaction between anatomical and non-anatomical factors. The main pathophysiological determinants include increased upper airway collapsibility, alterations in ventilatory control (loop gain), a reduced or increased arousal threshold, and an inadequate neuromuscular response of the pharyngeal dilator muscles. The dynamic combination of these factors determines the predisposition to pharyngeal collapse during sleep and the perpetuation of obstructive events [4]. The high prevalence of OSA in elderly populations may be attributable to the senescence mechanisms linked to aging, which involve the upper airway musculature and lead to increased collapsibility and poor neuromuscular response. Moreover, classical signs and symptoms of OSA such as excessive daytime sleepiness (EDS) may be subtle and atypical, or attributed to other geriatric conditions such as neurological diseases [5]. Recently, several studies have identified new distinct clusters of elderly OSA patients characterized by lower BMI and fewer symptoms compared to younger patients [6,7]. Taken together, all these findings support the existence of a specific age-related OSA phenotype. However, very elderly patients have been largely underrepresented in previous studies, and the clinical presentation of OSA in this population remains poorly characterized. In addition, most existing classifications of OSA phenotypes are based primarily on anthropometric variables, comorbidities, and sleepiness measured through the Epworth Sleepiness Scale (ESS), potentially failing to capture the full spectrum of daytime and nocturnal symptom variability across age groups and phenotypic clusters. Therefore, the aim of this study was to perform a cluster-based clinical phenotyping in a cohort of adult OSA patients, and to assess the potential association of these phenotypes with cognitive impairment, regardless of age.

## 2. Materials and Method

### 2.1. Study Design and Population

From January 2020 to July 2024, 409 patients aged 18 years or older and with moderate-to-severe OSA were enrolled after a one-night home sleep apnea test (HSAT) at the Sleep-Related Respiratory Disorders Ambulatory of the Geriatrics Unit, Renato Dulbecco Hospital—Mater Domini. Inclusion criteria were (i) age 18 years or older, (ii) a diagnosis of moderate-to-severe OSA, and (iii) the ability to provide informed consent. Exclusion criteria were (i) the presence of severe cognitive decline or dementia, (ii) the presence of psychiatric, neurological, or pain therapy that included medications that could alter OSA signs and symptoms (benzodiazepines, opioids, antiepileptic drugs, antipsychotics, etc.), (iii) a central or mixed hypopnea–apnea index greater than 4.9 e/h, and (iv) comorbidity with other sleep-related breathing disorders.

### 2.2. Clinical and Anthropometric Assessment

For each patient, demographic, anthropometric and medical data were collected. On the same day as the polygraphy, a complete medical history was obtained and OSA nocturnal and daytime symptoms were systematically assessed. The symptoms evaluated were snoring, apneas reported by the bed partner, nocturnal dyspnea, awakenings with the sensation of choking, nocturia, diaphoresis, daytime fatigue, subjective daytime sleepiness, attention or concentration difficulties, reduced manual dexterity, reduced libido, behavioral or personality changes, and morning headache. Daytime sleepiness was further assessed using the Epworth Sleepiness Scale (ESS). Cognitive status was assessed using the Montreal Cognitive Assessment (MoCA). Cognitive impairment (CoI) was classified according to established cut-off scores, with scores ≤ 25 considered pathological. Patients with a score lower than 18 were excluded [8].

### 2.3. Home Sleep Apnea Test

OSA diagnosis was established by a one-night home polygraphic recording using the SomnoTouch RESP device, equipped with a nasal cannula to assess the flow trace and snoring, two thoraco-abdominal belts to detect respiratory efforts, and a digital pulse oximeter for assessing peripheral arterial oxyhemoglobin saturation (SpO_2_), arterial pulse wave, and heart rate. A physician experienced in sleep disorders manually scored the recordings, in accordance with AASM recommendations. Sleep hypopnea was defined as a drop of ≥30% in breathing amplitude and in oxygen saturation ≥3%. Patients were considered healthy if AHI < 5 events/h, affected by mild OSA if AHI ranged between 5 and 14.9 events/h, by moderate OSA if AHI ranged between 15 and 29.9 events/h, and by severe OSA if AHI ≥ 30 events/h [9].

### 2.4. Statistical Analyses

Continuous data were expressed as mean ± standard deviation (SD). Normally distributed data were analyzed using the *t*-test for paired data; non-normally distributed data were analyzed using the Wilcoxon test. The Mann–Whitney test and Student’s *t*-test were performed for unpaired data; the chi-square test was performed when appropriate. Hierarchical cluster analysis was performed using Ward’s method to identify distinct phenotypes of OSA patients. The analysis included four clinically relevant variables: apnea–hypopnea index (AHI), percentage of time spent with oxygen saturation below 90% (T90), body mass index (BMI), and Epworth Sleepiness Scale (ESS). Prior to clustering, all variables were standardized (Z-scores) to account for differences in measurement scales and to ensure equal weighting in the clustering process. Ward’s method was chosen as it minimizes within-cluster variance and is widely used in clinical phenotyping studies. The optimal number of clusters was determined through visual inspection of the dendrogram and consideration of clinical interpretability of the resulting groups. This approach allowed the identification of three distinct and clinically meaningful clusters. Subsequently, univariable logistic regression models were constructed to investigate factors associated with the presence of cognitive impairment, evaluated as MoCA ≤ 25 pt. The relative odds ratio (OR) with the 95% confidence interval (95% CI) was calculated for each variable. The variables that significantly associated with the dependent variable were entered into a stepwise multivariable logistic regression model to evaluate the risk relationship that each variable has with the dependent variable. All values of *p* ≤ 0.05 were considered statistically significant. Statistical analysis was carried out using the SPSS V 20.0 program for Windows (SPSS Inc., Chicago, IL, USA).

## 3. Results

The study enrolled 409 patients, divided into three age groups (see Table 1). The first group included patients younger than 65 years (mean age 54.9 ± 8.9), the second group comprised elderly patients with ages between 65 and 74 (mean age 69.4 ± 2.9), and the third comprised very elderly patients (mean age 80.5 ± 4.69).

### 3.1. Demographic and Clinical Characteristics

In each group, the male sex was predominant. BMI significantly differed between groups, progressively decreasing in the group of elderly (31.0 ± 4.6) and very elderly (29.6 ± 5.8), compared with middle aged (33.4 ± 6.8) (*p* < 0.001). The number of comorbidities significantly increased with age (mean comorbidities in the middle aged group was 2.6 ± 1.6, in elderly 4.0 ± 2.0, and 5.2 ± 2.1 in very elderly; *p* < 0.001). The cardiovascular diseases were more prevalent in the very elderly and elderly group, especially arterial hypertension (74.8% vs. 86.2% vs. 88.1%: *p* = 0.007), carotid atherosclerosis (29% vs. 55.9% vs. 59.6%; *p* < 0.001), atrial fibrillation (7.1% vs. 13.1% vs. 22.9%; *p* = 0.001), prior cerebrovascular events (0.6% vs. 9.7% vs. 4.6%; *p* = 0.001), ischemic heart disease (IHD) (0.6% vs. 9.7% vs. 12.8%; *p* < 0.001), and heart failure (HF) (13.5% vs. 18.6% vs. 46.7%; *p* < 0.001), specifically in the mildly reduced (HFmrEF) and preserved (HFpEF) form. A similar trend in metabolic comorbidities was observed for dyslipidemia (56.8% vs. 71% vs. 71.6%; *p* = 0.011), while no significant difference was found in the prevalence of type 2 diabetes mellitus (T2DM) (36.8% vs. 44.1% vs. 49.5%; *p* = 0.109). Moreover, respiratory obstructive diseases such as COPD and asthma were more frequent in the elderly groups (12.3% vs. 23.4% vs. 33.9%; *p* < 0.001). Depression was similar between groups, while cognitive impairment was significantly more frequent in the very elderly group (4.5% vs. 9.7% vs. 45.9%; *p* < 0.001).

### 3.2. Polygraphic Characteristics and Symptomatology

Severe OSA represented the most frequent severity category across all the groups. No significant differences were observed between groups in the principal polygraphic parameters, including AHI, obstructive hypopnea index (oHI), oxygen desaturation index (ODI), T90, and mean SpO_2_ (see Table 2). Daytime sleepiness measured by ESS was slightly higher in the middle-aged group, although this difference did not reach statistical significance (8.1 ± 4.3 vs. 7.8 ± 4.2 vs. 7.8 ± 3.7; *p* = 0.814). Consistent differences in symptomatology between groups were found. Nocturnal symptoms were less frequent with increasing age, defining the very elderly group as “less symptomatic “than younger patients. Snoring was the most frequent symptom reported in all groups, although its prevalence progressively decreased in the elderly and very elderly groups compared with the middle-aged group (95.5% vs. 91.7% vs. 85.3%; *p* = 0.014). The second most common nocturnal symptom was witnessed apneas, which showed the same trend as snoring across age groups, although without reaching statistical significance (62.6% vs. 61.4% vs. 53.2; *p* = 0.271). Other nocturnal symptoms were less frequent, without significant difference across age, and were nocturnal chocking (26.5% vs. 18.6% vs. 16.5%; *p* = 0.101), nocturnal dyspnea (18.1% vs. 17.2% vs. 14.7%; *p* = 0.761), and nocturnal sweating (7.1% vs. 4.1% vs. 3.7%; *p* = 0.368). Nocturia did not differ between groups (29% vs. 24.1% vs. 22.9%; *p* = 0.467). Self-reported sleepiness was the most common daytime symptom in all groups; however, no statistically significant differences were observed between age groups, although the very elderly group reported this symptom less frequently (71.6% vs. 68.3% vs. 57.8%; *p* = 0.57). The second most prevalent daytime symptom referred was asthenia, and the same trend between groups as for sleepiness was observed (55.5% vs. 55.2% vs. 41.3; *p* = 0.042). Attention deficit and dexterity loss were more frequent in the elderly and very elderly groups, although without reaching statistical significance, while no difference was found regarding morning headache, reduced libido, and behavioral changes (see Table 2).

### 3.3. Cluster Analyses

**Figure 1:**
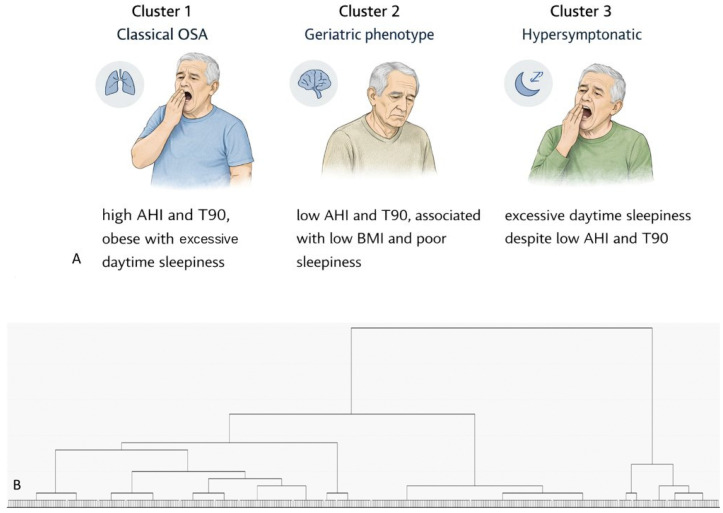
Clinical phenotypes of obstructive sleep apnea and hierarchical clustering analysis. (**A**) Schematic representation of the three clinical phenotypes identified through cluster analysis: Cluster 1 (classical OSA), characterized by typical features of obstructive sleep apnea; Cluster 2 (geriatric phenotype), characterized by lower symptom burden and a higher risk of cognitive impairment; and Cluster 3 (hypersymptomatic phenotype), characterized by marked daytime sleepiness despite lower disease severity. (**B**) Hierarchical clustering dendrogram (Ward’s method) based on standardized variables (AHI, T90, BMI, and ESS), illustrating the separation of the study population into three distinct clusters.

Cluster analysis revealed three distinct clinical phenotypes of OSA, according to different severity of AHI, BMI, nocturnal hypoxemia (T90), and excessive daytime symptoms, regardless of age group (Table 3). Cluster 1 represented the classical OSA phenotype. Patients with highest levels of AHI, BMI, and T90, and increased EDS characterized this cluster. Cluster 2 was characterized by the lowest levels of BMI and ESS, with mild severity of the polygraphic parameters. Cluster 3 showed dissociation between disease severity and symptoms burden, being characterized by patients reporting excessive sleepiness despite low AHI and T90, and preserved BMI (Figure 1A,B).

### 3.4. Demographic and Clinical Characteristics According to Cluster Group

The three clusters differed significantly in several demographic and clinical characteristics (Table 4). Cluster 2 comprised the oldest patients (63 ± 13.6 vs. 69.0 ± 10.7 vs. 67.1 ± 11.7; *p* < 0.001), while no significant difference was observed in sex distribution among clusters. Cardiovascular, metabolic, and respiratory disease prevalence was comparable across clusters, without statistically significant differences. Depressive disorder was most prevalent in Cluster 3 (11.1% vs. 17.4% vs. 23.2%; *p* = 0.48), while cognitive impairment was prevalent in Cluster 2 (8.1% vs. 34.2% vs. 14.2%; *p* < 0.001). Nocturnal symptoms were less frequent in Cluster 2, and despite snoring (92.9% vs. 92.3% vs. 89.7; *p* = 0.598) that was similar between cluster, apneas referred (66.7% vs. 45.8% vs. 69%; *p* < 0.001), and nocturnal chocking (28.3% vs. 15.5% vs. 21.9%; *p* = 0.048) were less common. Also, daytime symptoms showed a similar trend, with sleepiness (75.8% vs. 52.9% vs. 74.8%; *p* < 0.001) and asthenia (58.5% vs. 35.5% vs. 63.2%; *p* < 0.001) significantly less reported in Cluster 2. No other significant differences were found in symptoms.

### 3.5. Univariate and Multivariate Binary Logistic Regression Using Cognitive Impairment as Dependent Variable

Univariate and multivariate binary logistic regression analyses were performed to identify variables independently associated to cognitive impairment (Table 5). In the univariate analyses, age, male sex, and Cluster 2 were significantly associated with cognitive impairment. The multivariate analyses confirmed age (OR: 1.139; C.I.: 1.099–1.179; *p* < 0.001), male sex (OR: 2.407; C.I.: 1.197–4.838 *p* = 0.014), and belonging to Cluster 2 (OR: 4.175; C.I.: 2.329–7.484; *p* < 0.001) as variables independently associated to a higher risk of cognitive impairment. Cluster 3 showed a protective trend against cognitive decline. In the univariate analysis, belonging to Cluster 3 was associated with lower odds of cognitive decline (OR 0.523, 95% CI 0.306–0.894). This association remained significant after multivariate adjustment (OR 0.459, 95% CI 0.251–0.839), suggesting that subjects in Cluster 3 had a reduced likelihood of cognitive impairment compared with the reference group.

## 4. Discussion

This study provided further details on the phenotyping of Obstructive Sleep Apnea Syndrome (OSAS) independently of classic polygraphic parameters and age. Three distinct OSAS clusters were identified among patients with comparable disease severity. This approach may help identify novel clinical presentations of OSAS, especially in elderly patients, who are predominantly represented in Cluster 2 in this study. In our study, elderly patients showed significantly lower BMI, suggesting that non-anatomical rather than anatomical factors are the determinant in the pathophysiology of upper airway collapse. Previous studies have already shown that older OSA patients had lower BMI compared to younger patients [10], and distinct pathophysiological mechanisms are associated with aging, including a pronounced susceptibility to upper airway collapse [11], probably due to fat deposition in the pharyngeal muscles [12] and nocturnal rostral fluid shift [13].

The burden of comorbidities is also higher in older groups, especially in neurological, cardiovascular, metabolic and respiratory diseases. Notably, there were no significant differences in the prevalence of T2DM, probably due to a higher prevalence in the younger group and a reduced prevalence in the elderly. A substantial body of evidence supports a bidirectional relationship between comorbidities and OSA. A hallmark of aging is the increased comorbidity burden, especially cardiovascular diseases such as arterial hypertension, atrial fibrillation, and heart failure. The bidirectional relationship explains that OSA is an independent risk factor for arterial hypertension and atrial fibrillation, while heart failure may predispose to OSA through increased susceptibility to the upper airway collapse due to the rostral shift, as observed in other conditions associated with fluid retention such as chronic kidney disease [14]. These findings suggest that older patients, characterized by several comorbidities, are exposed to an augmented risk of OSA. Furthermore, we found a significant prevalence of cognitive impairment in the very elderly group. OSA may contribute to cognitive impairment through different mechanisms, including intermittent hypoxemia and changes in sleep macro- and microarchitecture [15].

Our study demonstrated a clear trend in OSA symptoms across aging. Older groups showed fewer symptoms, both daytime and nocturnal. Although nocturnal symptoms were the most frequently reported in the elderly and very elderly groups, they were significantly less prevalent compared to the younger group. OSA in older patients is “less symptomatic” and less overtly recognized. Daytime symptoms showed the same trend; ESS scores were lower with aging, as were reported sleepiness and asthenia, while cognitive symptoms were more prevalent in older patients. These findings are consistent with existing literature. Ida et al. [10] and Monti et al. [16] similarly reported a lower prevalence of nocturnal symptoms such as snoring and witnessed apneas in older age groups. Geriatric patients may report less daytime sleepiness and asthenia because of reduced social productivity demands, and the opportunity to take naps during the day. Moreover, the ESS has limited diagnostic performance in older patients, because older subjects have difficulty responding to all ESS items, with only one patient in four complaining of EDS having an abnormal ESS score [17]. Furthermore, an important aspect to consider is the potential bidirectional relationship between cognitive impairment and symptom reporting. In older adults, early cognitive decline may impair awareness, perception, and verbalization of symptoms such as daytime sleepiness and fatigue. Consequently, lower ESS scores may reflect underreporting rather than a true absence of symptoms.

In order to identify distinct OSA phenotypes regardless of age group, we performed a cluster analysis using the variables AHI, T90, BMI, and ESS, identifying three distinct phenotypes. Cluster 1 represents the classical OSA phenotype, characterized by high AHI, T90, BMI, and ESS. This cluster has a moderate mean age and a well-represented profile of nocturnal and daytime symptoms, as well as cardiovascular, respiratory, and metabolic diseases. In contrast, this is the cluster with the lowest prevalence of cognitive impairment. Cluster 2 exhibits the lowest AHI, T90, BMI, and ESS. This cluster may be regarded as representative of the geriatric phenotype, as it comprises the oldest patients, and is characterized by a significantly lower prevalence of nocturnal and daytime symptoms, while the prevalence of cognitive impairment is the highest.

A key point that deserves clarification is the definition of Cluster 2 as a “geriatric phenotype.” While this cluster showed the highest mean age among the identified groups, age alone does not fully explain its characterization. Indeed, all clusters included a substantial proportion of individuals aged over 65 years, and the overlap in age distribution limits the clinical interpretability of chronological age as the sole defining feature. Rather, Cluster 2 appears to represent a multidimensional phenotype consistent with previously described features of OSA in older adults. Specifically, it is characterized by lower BMI, reduced nocturnal and daytime symptom burden, and lower subjective sleepiness, all of which have been reported as typical features of OSA in aging populations [5,6]. In addition, this cluster showed the highest prevalence of cognitive impairment and was independently associated with cognitive decline in multivariable analysis. Taken together, these findings suggest that the term “geriatric phenotype” should be interpreted not merely as an age-defined category, but as a clinical construct reflecting the interaction between aging-related physiological changes, atypical symptom presentation, and increased vulnerability to cognitive impairment. This interpretation is in line with emerging evidence that OSA in older adults may present with less overt symptoms but greater impact on neurocognitive outcomes.

Cluster 3 represents an excessively symptomatic phenotype despite low AHI and T90. Asthenia and sleepiness are the symptoms most frequently reported compared to the other two clusters, and depressive mood disorder is significantly more prevalent in this phenotype.

Previous studies investigated the heterogeneity and complexity of OSA through cluster analyses. Ye et al. firstly identified three clusters of OSA patients based on reported symptoms: the “disturbed sleep”, “minimally symptomatic”, and “excessive daytime sleepiness” phenotypes [18]. Subsequently, Keenan et al. proposed a similar cluster analysis and identified five clusters: “labeled disturbed sleep, minimal symptoms” and “upper airway symptoms with sleepiness”, “labeled upper airway symptoms dominant” and “sleepiness dominant” [19]. Building on these pioneering studies, subsequent authors have expanded this field, including analyses in patients aged 65 years or older [6,20]. However, some differences between our results and the above studies should be highlighted. First of all, the above studies used exclusively symptoms to differentiate the clusters, while we used anthropometric data (BMI), polygraphic indices (AHI, T90), and sleepiness through the ESS. Our Cluster 1, the “sleepy obese” OSA phenotype, is recognizable across prior classification systems, as it represents the classical phenotype. Cluster 2, the geriatric cluster, is a relatively novel phenotype that does not correspond to any previously described category. The above studies did not include a representative number of very elderly patients, and typical geriatric conditions, such as mood disorders and cognitive impairment, were not investigated. This geriatric phenotype raises new questions as to whether frailty, more than aging, may be involved in the mechanisms responsible for upper airway collapse. Similarly, the phenotype of excessive symptoms despite low AHI has not been described in other classification systems, although its description resembles the phenotype “upper airway symptoms with sleepiness” described by Keenan et al. [19].

Subsequently, we performed a binary logistic regression using the presence of cognitive impairment as the dependent variable, in order to identify independent variables associated with an elevated risk of this important marker of frailty in a cohort of adult patients affected by OSA. As expected, aging and male sex were associated with cognitive impairment [21,22], but the variable that showed the strongest association was the geriatric phenotype (Cluster 2).

This study has several limitations. First, its cross-sectional design precludes causal inference, and results are not comparable to those of a randomized controlled trial (RCT). The imbalance in the number of patients between men and women is an inherent limitation of our study, as the ideal ratio between the two groups would have been 1:1 (instead of 2:1). The predominance of male participants may have influenced the identified cluster structure, as sex-related differences in OSA presentation are well recognized. Women with OSA often report less typical symptoms and may be underdiagnosed or present with different clinical profiles [23], which could not be fully captured in our analysis. In addition, patients with severe cognitive impairment were excluded at study entry in order to ensure the reliability of clinical assessment and questionnaire-based measures. While this approach was methodologically necessary, it may have led to an underestimation of the overall burden and severity of cognitive dysfunction in OSA. Consequently, our findings likely represent only a partial view of the relationship between OSA and cognitive impairment, particularly in older adults, where more advanced stages of cognitive decline are common. Furthermore, the lack of data on MoCA trajectory and follow-up represents another limitation of the study. Therefore, this study stands as a hypothesis generator.

However, this study also has some strength: it is a study with a large sample size which enrolled elderly patients suffering from numerous comorbidities who are often excluded from RCTs.

## 5. Conclusions

This observational real-life study provides a phenotyping of the adult OSAS patient population, regardless of age and OSAS severity. Despite comparable disease severity, three clusters were identified, the second of which is representative of the elderly OSAS population, frequently underrepresented in RCTs. In particular, in this cluster, patients were older and had lower BMI, a reduced prevalence of classic nocturnal symptoms, lower reported daytime sleepiness and asthenia, higher burden of comorbidities, and significantly higher prevalence of cognitive impairment. These results suggest that aging significantly alters the clinical presentations of OSAS. Moreover, as a further confirmation, Cluster 2 is associated with the elderly population; this cluster confers an approximately three-fold increased risk of CoI.

## Figures and Tables

**Table 1:** Demographic and clinical characteristics of the study population according to age group.

	All Population (*n* 409)	≤64 Years (*n* 155)	65–74 Years (*n* 145)	≥75 Years (*n* 109)	*p*
Age, years	66.9 ± 12.0	54.9 ± 8.9	69.4 ± 2.9	80.5 ± 4.6	<0.001 ^a^
Sex, w/m (%)	119/290 (29.1/70.9)	44/111 (28.4/71.6)	47/98 (32.4/67.6)	28/81 (25.7/74.3)	0.490
BMI, kg/m^2^	31.5 ± 6.0	33.4 ± 6.8	31.0 ± 4.6	29.6 ± 5.8	<0.001 ^b^
Moderate OSA, n (%)	126 (30.8)	47 (30.3)	39 (26.9)	40 (36.7)	0.243
Severe OSA, n (%)	283 (69.2)	108 (69.7)	106 (73.1)	69 (63.3)	0.243
HTN, n (%)	337 (82.4)	116 (74.8)	125 (86.2)	96 (88.1)	0.007
T2DM, n (%)	175 (42.8)	57 (36.8)	64 (44.1)	54 (49.5)	0.109
Dyslipidaemia, n (%)	269 (65.8)	88 (56.8)	103 (71)	78 (71.6)	0.011
Carotid ATS, n (%)	191 (46.7)	45 (29)	81 (55.9)	65 (59.6)	<0.001
IHD, n (%)	29 (7.1)	1 (0.6)	14 (9.7)	14 (12.8)	<0.001
HF, n (%)	96 (23.4)	18 (13.5)	27 (18.6)	51 (46.7)	<0.001
HFrEF, n (%)	30 (7.3)	8 (5.2)	11 (7.6)	11 (10.1)	0.315
HFmrEF, n (%)	20 (4.9)	5 (3.2)	3 (2.1)	12 (11)	0.002
HFpEF, n (%)	46 (11.2)	5 (3.2)	13 (9)	28 (25.7)	<0.001
Atrial Fibrillation, n (%)	55 (13.4)	11 (7.1)	19 (13.1)	25 (22.9)	0.001
PM/ICD, n (%)	19 (4.6)	3 (1.9)	6 (4.1)	10 (9.2)	0.021
TIA/Stroke, n (%)	20 (4.9)	1 (0.6)	14 (9.7)	5 (4.6)	0.001
COPD/Asthma, n (%)	90 (22)	19 (12.3)	34 (23.4)	37 (33.9)	<0.001
NRI, n (%)	118 (28.9)	46 (29.7)	46 (31.7)	26 (23.9)	0.375
CKD, n (%)	60 (14.7)	3 (1.9)	15 (10.3)	42 (38.5)	<0.001
Smokers, n (%)	34 (8.3)	14 (9)	17 (11.7)	3 (2.8)	0.034
Ex Smokers, n (%)	38 (9.3)	9 (5.8)	15 (10.3)	14 (12.8)	0.132
Depression. n (%)	74 (18.1)	28 (18.1)	28 (19.3)	18 (16.5)	0.849
CoI, n (%)	83 (20.3)	11 (7.1)	16 (11)	56 (45.9)	<0.001
Comorbidities, n	3.8 ± 2.1	2.6 ± 1.7	4.0 ± 2.0	5.2 ± 2.1	<0.001 ^a^

**Abbreviations:** BMI: Body Mass Index; OSA: obstructive sleep apnea; HTN: arterial hypertension; T2DM: type 2 diabetes mellitus; Carotid ATS: carotid atherosclerosis; IHD: ischaemic heart disease; HF: heart failure; HFrEF: heart failure with reduced ejection fraction; HFmrEF: heart failure with mildly reduced ejection fraction; HFpEF: heart failure with preserved ejection fraction; PM/ICD: pace maker/implantable cardioverter defibrillator; TIA: transient ischemic attack; COPD: chronic obstructive pulmonary disease; NRI: nocturnal respiratory insufficiency; CKD chronic kidney disease, CoI: cognitive impairment. Post hoc Bonferroni analyses: ^a^: Group 1 vs. 2 *p* < 0.001, Group 1 vs. 3 *p* < 0.001, Group 2 vs. 3 *p* < 0.0001. ^b^: Group 1 vs. 2 *p* = 0.002, Group 1 vs. 3 *p* < 0.001, Group 2 vs. 3 *p* = 0.213.

**Table 2:** Polygraphic characteristics and symptomatology of the study population according to age group.

	All Population (*n* 409)	≤64 Years (*n* 155)	65–74 Years (*n* 145)	≥75 Years (*n* 109)	*p*
AHI, e/h	43.0 ± 21.8	45.1 ± 24.6	43.5 ± 20.1	39.2 ± 19.2	0.085
OAI. e/h	24.4 ± 21.3	27.0 ± 24.5	23.3 ± 19.4	22.2 ± 18.2	0.145
OHI, e/h	15.1 ± 13.8	14.3 ± 12.4	17 ± 15.5	13.9 ± 12.9	0.136
ODI, e/h	43.0 ± 22.5	44.5 ± 24.7	44.2 ± 20.5	39.4 ± 21.5	0.135
T90, %	21.3 ± 24.4	19.8 ± 21.8	24.6 ± 26.1	19.2 ± 25.3	0.137
SpO_2_, (%)	91.8 ± 3.1	91.8 ± 2.8	91.6 ± 3.2	91.9 ± 3.2	0.760
ESS, pt	7.9 ± 4.1	8.1 ± 4.3	7.8 ± 4.2	7.8 ± 3.7	0.814
Snoring, n (%)	374 (91.4)	148 (95.5)	133 (91.7)	93 (85.3)	0.014
Apneas referred, n (%)	244 (59.7)	97 (62.6)	89 (61.4)	58 (53.2)	0.271
Nocturnal dyspnea, n (%)	69 (16.9)	28 (18.1)	25 (17.2)	16 (14.7)	0.761
Nocturnal chocking, n (%)	86 (21)	41 (26.5)	27 (18.6)	18 (16.5)	0.101
Nocturia, n (%)	105 (25.7)	45 (29)	35 (24.1)	25 (22.9)	0.467
Nocturnal sweating, n (%)	21 (5.1)	11 (7.1)	6 (4.1)	4 (3.7)	0.368
Asthenia, n (%)	211 (51.6)	86 (55.5)	80 (55.2)	45 (41.3)	0.042
Sleepiness, n (%)	273 (66.7)	111 (71.6)	99 (68.3)	63 (57.8)	0.057
Attention deficit, n (%)	94 (23)	30 (19.4)	38 (26.2)	26 (23.9)	0.359
Dexterity loss, n (%)	21 (5.1)	5 (3.8)	6 (4.1)	10 (9.2)	0.078
Reduced libido, n (%)	17 (4.2)	5 (3.2)	9 (6.2)	3 (2.8)	0.300
Morning headache, n (%)	52 (12.7)	24 (15.5)	15 (10.3)	13 (11.9)	0.393
Behavioral changes, n (%)	8 (2)	1 (0.6)	5 (3.4)	2 (0.5)	0.214

**Abbreviations:** AHI: Apnea Hypopnea Index; OAI: Obstructive Apnea Index, OHI: Obstructive Hypopnea Index, ODI: Oxygen Desaturation Index, T90: percentual time oxygen saturation under 90%, ESS: Epworth Sleepiness Scale.

**Table 3:** Cluster analyses.

	Cluster 1 (*n* 99)	Cluster 2 (*n* 155)	Cluster 3 (*n* 155)	*p*
**ZAHI**	**1.07**	**−0.17**	**−0.51**	**<0.001**
	**High**	**Low**	**Low**	
**ZT90**	**1.19**	**−0.23**	**−0.53**	**<0.001**
	**High**	**Low**	**Low**	
**ZBMI**	**0.79**	**−0.53**	**0.02**	**<0.001**
	**High**	**Low**	**Mild**	
**ESS**	**0.52**	**−0.87**	**0.53**	**<0.001**
	**High**	**Low**	**High**	

Cluster analyses identifying three distinct patient phenotypes based on standardized (Z-score) values of AHI, nocturnal hypoxemia (TC90), BMI, and daytime sleepiness evaluated by Epworth Sleepiness Scale (ESS) and are reported in the table with different colors (Pink: high z-scores; blue: low z-scores; orange: moderate z-scores.). Values are expressed as standardized scores (Z-scores), allowing direct comparison across different scales. Positive values indicate values above the sample mean, while negative values indicate values below the mean. Cluster 1 is characterized by high values across all variables, Cluster 2 by consistently low values, and Cluster 3 by low AHI and TC90, intermediate BMI, and high ESS scores. **Abbreviations:** AHI: Apnea Hypopnea Index; T90: percentual time oxygen saturation under 90%; BMI: Body Mass Index; ESS: Epworth Sleepiness Scale.

**Table 4:** Clinical characteristics according to cluster group.

	Cluster 1 (*n* 99)	Cluster 2 (*n* 155)	Cluster 3 (*n* 155)	*p*
Age, years	63 ± 13.6	69.0 ± 10.7	67.1 ± 11.7	<0.001 ^a^
Sex, w/m (%)	24/75 (24.2/75.8)	41/114 (26.5/73.5)	54/101 (34.8/65.2)	0.127
AHI, e/h	66.3 ± 24.0	39.2 ± 16.0	31.8 ± 12.1	<0.001 ^a^
TC90, %	50.4 ± 25.6	15.8 ± 17.1	8.3 ± 11.1	<0.001 ^a^
BMI, kg/m^2^	36.3 ± 6.3	28.3 ± 4.2	31.7 ± 5.4	<0.001 ^a^
ESS, n	10.0 ± 4.1	4.3 ± 1.9	10.1 ± 3.1	<0.001 ^a^
HTN, n (%)	78 (78.8)	136 (87.7)	123 (79.4)	0.085
T2DM, n (%)	48 (48.5)	60 (38.7)	67 (43.2)	0.305
Dyslipidaemia, n (%)	62 (62.6)	111 (71.6)	96 (65.8)	0.150
IHD, n (%)	9 (9.1)	10 (6.5)	10 (6.5)	0.673
HF, n (%)	28 (28.2)	34 (21.9)	34 (21.9)	0.346
Atrial Fibrillation, n (%)	11 (11.1)	23 (14.8)	21 (13.5)	0.696
TIA/Stroke, n (%)	6 (6.1)	8 (5.2)	6 (3.9)	0.718
COPD, n (%)	27 (27.3)	30 (19.4)	33 (21.3)	0.320
CKD, n (%)	17 (17.2)	25 (16.1)	18 (11.6)	0.384
Depression. n (%)	11 (11.1)	27 (17.4)	36 (23.2)	0.48
CoI, n (%)	8 (8.1)	53 (34.2)	22 (14.2)	<0.001
Snoring, n (%)	92 (92.9)	143 (92.3)	139 (89.7)	0.598
Apneas referred, n (%)	66 (66.7)	71 (45.8)	107 (69)	<0.001
Nocturnal dyspnea, n (%)	22 (22.2)	22 (14.2)	25 (16.1)	0.238
Nocturnal chocking, n (%)	28 (28.3)	24 (15.5)	34 (21.9)	0.048
Nocturia, n (%)	28 (28.3)	33 (21.3)	44 (28.4)	0.285
Nocturnal sweating, n (%)	3 (3)	7 (4.5)	11 (7.1)	0.325
Asthenia, n (%)	58 (58.5)	55 (35.5)	98 (63.2)	<0.001
Sleepiness, n (%)	75 (75.8)	82 (52.9)	116 (74.8)	<0.001
Attention deficit, n (%)	25 (25.3)	30 (19.4)	39 (25.2)	0.395
Dexterity loss, n (%)	3 (3)	9 (5.8)	9 (5.8)	0.552
Reduced libido, n (%)	3 (3)	5 (3.2)	9 (5.8)	0.425
Morning headache, n (%)	15 (15.2)	18 (11.6)	19 (12.3)	0.695
Behavioral changes, n (%)	4 (4)	2 (1.3)	2 (1.3)	0.228

Abbreviations: AHI: Apnea Hypopnea Index; T90: percentual time oxygen saturation under 90%; BMI: Body Mass Index; ESS: Epworth Sleepiness Scale; HTN: arterial hypertension; T2DM: type 2 diabetes mellitus; IHD: ischaemic heart disease; HF: heart failure; TIA: transient ischemic attack; COPD: chronic obstructive pulmonary disease; CKD chronic kidney disease, CoI: cognitive impairment. Post hoc Bonferroni analyses: ^a^: Group 1 vs. 2 *p* = 0.001, Group 1 vs. 3 *p* = 0.041, Group 2 vs. 3 *p* = 0.529.

**Table 5:** Univariate and multivariate binary logistic regression using cognitive impairment as dependent variable.

	OR	C.I. 95%	*p*	OR	C.I. 95%	*p*
Age, 1 year increase	1.143	1.102–1.186	<0.001	1.139	1.099–1.179	<0.001
BMI ≥ 30 kg/m^2^, y/n	1.031	0.969–1.097	0.331			
Male Sex, y/n	2.480	1.222–5.032	0.012	2.407	1.197–4.838	0.014
Asthenia, y/n	0.732	0.361–1.482	0.385			
Sleepiness, y/n	1.296	0.634–2.646	0.477			
Cluster 1, y/n	0.503	0.188–1.343	0.170			
Cluster 2, y/n	3.794	1.914–7.520	<0.001	4.175	2.329–7.484	<0.001
Cluster 3, y/n	0.523	0.306–0.894	0.18	0.459	0.251–0.839	0.11

On the left is reported the univariate binary logistic regression, while on the right the multivariate logistic regression assessing factors associated with cognitive impairment. In multivariate analyses, increasing age, male sex, and belonging to Cluster 2 were independently associated with a higher risk of cognitive impairment.

## Data Availability

Data available on request. The data underlying this article will be shared on reasonable request to the corresponding author.
